# A Perspective Review: Analyzing Collagen Alterations in Ovarian Cancer by High-Resolution Optical Microscopy

**DOI:** 10.3390/cancers16081560

**Published:** 2024-04-19

**Authors:** Kristal L. Gant, Manish S. Patankar, Paul J. Campagnola

**Affiliations:** 1 Department of Obstetrics and Gynecology, University of Wisconsin-Madison, Madison, WI 53706, USA; kgant@wisc.edu; 2 Department of Biomedical Engineering, University of Wisconsin-Madison, Madison, WI 53706, USA; 3 Carbone Cancer Center, University of Wisconsin-Madison, Madison, WI 53706, USA

**Keywords:** high-grade serous ovarian cancer, ECM alterations, collagen reorganization, clinical interventions

## Abstract

**Simple Summary:**

The perspective of this paper will focus on high-resolution imaging of collagen alterations in high-grade serous ovarian cancer (HGSOC). We will highlight the use of the collagen-specific/sensitive Second Harmonic Generation (SHG) microscopy in delineating changes across multiple size scales between HGSOC, other ovarian tumors, HGSOC precursors and normal tissues. Specifically, we utilize machine learning techniques to differentiate these tissues with high accuracy and further exploit the underlying SHG physics to determine sub-resolution information on fibril assembly and macro/supramolecular collagen structure. We also describe how SHG can be combined with other modalities including fluorescence and optical coherence tomography (OCT). Lastly, we discuss challenges and opportunities for translation to in vivo applications, focusing on advances in endoscopic technology. We postulate that successful diagnosis/treatment of HGSOC requires an integrated approach of ex vivo microscopic and molecular analyses to establish the foundation for in vivo imaging.

**Abstract:**

High-grade serous ovarian cancer (HGSOC) is the predominant subtype of ovarian cancer (OC), occurring in more than 80% of patients diagnosed with this malignancy. Histological and genetic analysis have confirmed the secretory epithelial of the fallopian tube (FT) as a major site of origin of HGSOC. Although there have been significant strides in our understanding of this disease, early stage detection and diagnosis are still rare. Current clinical imaging modalities lack the ability to detect early stage pathogenesis in the fallopian tubes and the ovaries. However, there are several microscopic imaging techniques used to analyze the structural modifications in the extracellular matrix (ECM) protein collagen in ex vivo FT and ovarian tissues that potentially can be modified to fit the clinical setting. In this perspective, we evaluate and compare the myriad of optical tools available to visualize these alterations and the invaluable insights these data provide on HGSOC initiation. We also discuss the clinical implications of these findings and how these data may help novel tools for early diagnosis of HGSOC.

## 1. Introduction

**Figure 1:**
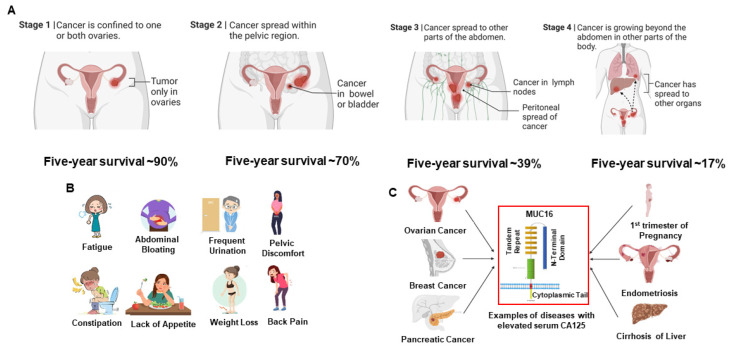
High-grade serous ovarian cancer (HGSOC) is a difficult disease to diagnose and treat. (**A**) Surgical staging of ovarian cancer correlates with the prognosis of patients to conventional debulking surgery combined with platinum and taxol-based chemotherapy. (**B**) Most women with HGSOC experience relatively common symptoms, which, by themselves, are insufficient for cancer diagnosis. (**C**) Serum levels of CA125, a repeating peptide epitope of the large molecular weight mucin, MUC16, are elevated in most patients with HGSOC. Elevation of CA125 is also observed in other cancers, as well as benign conditions. Therefore, while the FDA-approved serum CA125 test is useful in monitoring the recurrence of HGSOC in women already diagnosed with this cancer, this test is not used as a screening tool for early detection of ovarian cancer.

In 2023, an estimated 19,710 and 13,270 women will be diagnosed with and will die from ovarian cancer in the United States, respectively. Ovarian cancer is the fifth leading cause of death among all cancers in women and is the #1 cause of death among gynecological cancers. The five-year survival rates of patients depend on the extent of the spread of the cancer beyond the ovaries and to the peritoneal organs. In early stages (stages Iand II, Figure 1) [1], when the cancer is confined to the ovarian surface, cytoreductive surgery combined with chemotherapy results in maximum benefit to patients with five-year survival observed in ~90% of the cases. However, in the majority of the patients (70% or more), ovarian cancer is detected at an advanced stage (stages III or IV, Figure 1) [1]) with the tumors already metastasized to the serosal surface and organs in the peritoneal cavity. While most patients with such advanced disease initially respond to debulking surgery and platinum and taxol-based chemotherapy, recurrence of the tumor is often observed, resulting in significantly lower five-year survival (~30%). Even with the development of modern clinical approaches and targeted and biologic therapies (PARP inhibitors and immunotherapies, for example), these statistics have remained unchanged over the past several decades. Developing new strategies for early detection and treatment of high-grade serous ovarian cancer is therefore a major unmet need for effective management of this cancer. With worldwide estimates of 314,000 new cases and 207,000 deaths per year, ovarian cancer is a major health problem for women across all continents [2].

Figure 1 shows the staging and commonly associated symptoms of HGSOC. Early stage disease is often associated with bloating, vaginal bleeding and other clinical symptoms that are not truly indicative of cancer in the gynecologic tract (Figure 1B). The lack of specific clinical symptoms combined with the low sensitivity and specificity of the currently used biomarker, CA125 (Figure 1C) results in HGSOC being detected at an advanced stage in most patients. Imaging modalities (e.g., ultrasound, MRI, CT, PET) also do not have sufficient resolution or sensitivity to meet this early detection challenge and also lack sufficient specificity for discriminating subtypes of ovarian cancer [3,4,5,6,7,8]. Only a fortuitous clinical examination by trained clinicians and patients who are vigilant about the seemingly vague symptoms generally leads to early detection (stages I or II) of HGSOC. 

Similar to most epithelial cancers, HGSOC is characterized by early changes in the extracellular matrix (ECM) within the tumor microenvironment [9,10]. However, unlike most tumors that largely metastasize via the blood and lymphatic systems, the primary mechanism in HGSOC is the exfoliation of cancer cells from primary lesions that are transported via ascites and can re-attach in the peritoneum. The ECM on the surfaces of the ovaries and peritoneal organs plays an important role in promoting attachment and metastasis of HGSOC [11]. Therefore, studying these corresponding cell–ECM interactions at the microscopic level in both primary and metastatic sites is key to understanding the progress of disease. As adhesion and migration are in part governed by the collagen fiber morphology, probing this structure is a potentially powerful diagnostic/prognostic approach. This was first conclusively demonstrated by Keely and co-workers who, in a series of seminal papers, showed that different stages of breast tumors had characteristic collagen fiber alignment, and, moreover, that these patterns were prognostic of survival [12,13,14]. Collagen fiber patterns have also now been used to discriminate normal and malignant tissues in several tissues [14,15,16], including those of the fallopian tubes and ovaries, as noted below. 

**Figure 2:**
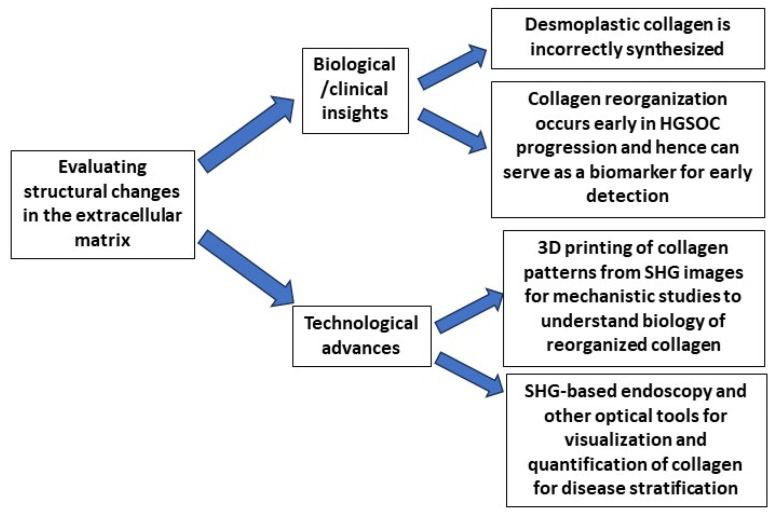
Evaluation of the extracellular matrix surrounding ovarian tumors is providing new insights into the biology of cancer and new technologies to understand the importance of altered collagen architecture in HGSOC progression. SHG imaging of ovarian tumors has demonstrated major alterations in the collagen architecture. Our recent studies have shown that these changes in collagen occur early and can even be detected in the precursor lesion (serous tubal intraepithelial carcinoma, STIC) of HGSOC. These changes can serve as biomarkers for the disease. Our group has now developed a 3D printing approach to make mimics of the collagen based on normal and HGSOC tissues. These 3D patterns have allowed us to demonstrate that cancer-associated collagen promotes the migration of ovarian cancer cells. Finally, the demonstration that collagen is altered early in the progression of HGSOC suggests that SHG-based endoscopic or other optical methods can be developed for in vivo or ex vivo monitoring of tissues for the detection of HGSOC signatures.

Our recent analyses of collagen structure in ex vivo human HGSOC [17,18] and its precursor lesions [19,20] using high-resolution optical microscopy are pointing to a new direction for the possibility of early detection of this cancer. Here, we will highlight our use of the collagen-specific/sensitive Second Harmonic Generation (SHG) microscopy in delineating changes across multiple size scales between HGSOC, other ovarian tumors, HGSOC precursors and normal tissues. Specifically, we utilize machine learning techniques to differentiate these tissues with high accuracy and further exploit the underlying SHG physics to determine sub-resolution information on fibril assembly and macro/supramolecular collagen structure. This overall direction is depicted in Figure 2. We note that SHG studies provide more structural information than possible by standard pathology but can often be carried out on the same slide. We further provide our perspective on the high-resolution optical imaging modalities (separately and in conjunction with SHG) that can be developed for clinical staging and early detection of HGSOC, including multiphoton microscopy and optical coherence tomography-based optical scattering measurements and potentially superior monitoring of treatment efficacy.

## 2. Imaging Extracellular Matrix ECM Alterations in Ovarian Cancer

### 2.1. Second Harmonic Generation (SHG) Microscopy of Ex Vivo Tissues

SHG is a nonlinear coherent technique that upconverts two lower-energy photons into a photon with twice the frequency and half the wavelength [21]. The physical constraints require a non-centrosymmetric (lacking a center of symmetry) environment, on the size scale of the λ_SHG_ or about 500 nm, where the organization of fibrils/fibers determine emission characteristics [22] and, as shown below, can be exploited to extract macro/supramolecular information [23], as well as fiber morphology [18]. We will describe how all these attributes are different in HGSOC and other ovarian tumors relative to normal stroma. Additionally, the modality is label-free and can image through several hundred microns of highly scattering dense collagen. Lastly, we note that SHG more clearly visualizes collagen fibers better than H&E histology or Picrosirius Red polarization microscopy.

#### 2.1.1. Collagen Fiber Morphology

##### Tumors in the Ovary

**Figure 3:**
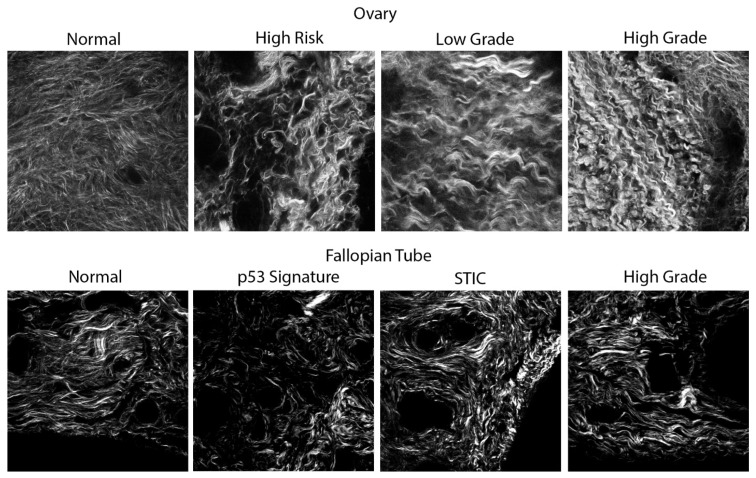
(**Top row**): Representative single SHG optical sections of a spectrum of ovarian tissues obtained from the ovarian cortex. (**Bottom row**): Representative single SHG optical sections of a spectrum of HGSOC tissues from the fallopian tube precursors. Field size = 170 × 170 microns for all images. The clinical status of all samples used in this study was examined by pathologists who are trained in gynecologic pathology and who used clinically approved practices (hematoxylin staining and immunohistochemistry) to identify the tissues.

There now have been several reports by several labs using SHG to image human and mouse ovarian cancers [24,25,26,27]. In our research, we have shown that normal stroma, high-risk stroma (stroma surrounding the serous tubal intraepithelial carcinoma, the precursor lesions of HGSOC) and low-grade serous, endometrioid, benign and HGSOC tumors each have a characteristic fiber pattern [18]. Representative images are shown for these classes in the top row of Figure 3. For example, in normal tissues, the collagen fibers appear cross-hatched and interwoven and are arranged in no apparent order. However, in HGSOC tissue, the collagen fibers have a highly characteristic sine wave pattern and are aligned over up to a few hundred microns in length. To characterize these differences, using machine learning, we used a novel form of texture analysis coupled with machine learning as a classification algorithm to differentiate between the six classes noted above. Here, we used a method known as “textons” [28], which does not rely on simple features such as length, width and alignment, but identifies features based on convolution with a 38-element basis set. We developed this approach for both 2D [17] and 3D [18] and found significantly improved accuracies in the latter embodiment (~15–20%). We suggested that this arose because the intrinsic heterogeneity was better sampled. The texton method is quite powerful because it is based on substantial data sets of several hundred images per class.

##### Collagen Reorganization in Early HGSOC Precursor Lesions

It is now well documented that most HGSOC tumors originate in the fallopian tubes (FT) from two precursor lesions: p53 signatures and serous tubal intraepithelial carcinoma (STIC) [10,29]. The ovarian surface is the first site for metastasis of the HGSOC that develops from these two precursors. We have now performed SHG imaging on STICs and p53 precursors, that were occurring concurrently with HGSOC tissues [19], where representative images are shown in the bottom row of Figure 3. Here, we developed a linear discriminant based on multivariate variables derived from grey-level co-occurrence matrix texture features, FFT features and fiber morphologies derived from CT-FIRE [30]. We achieved a classification accuracy of >95% for HGSOC vs. the others and a more modest ~75% between STIC and normal regions. Interestingly, the collagen fibers in the high-grade tumors in the FT (highly aligned wavy patterns) strongly resembled that of HGSOC in the ovary itself, further supporting the idea of the FT as the origin site. We further conducted analogous SHG imaging and classification on pure precursor lesions that did not have a concurrent HGSOC lesion and found that there were subtle changes in collagen organization in p53 signatures and STIC lesions that were distinct from each other and normal regions [20]. Notably, the collagen appears relatively normal within a couple of hundred microns from STIC regions. Collectively, these studies strongly suggest that collagen changes occur early in HGSOC development and, moreover, that these can be detected by SHG.

#### 2.1.2. Analysis of Sub-Resolution Features

Collagen has a highly conserved hierarchical organization with many ranges of size scales, beginning with the single α-chains, which are hydrogen bonded into the triple helix (~300 nm × 1 nm), where these are covalently crosslinked into fibrils (~50 nm in diameter), which are crosslinked into fibers (~1-micron diameter) and are the quantity viewed in the SHG microscope. However, we can extract information on several of these sub-resolution aspects through a combination of experimental and computational/theoretical approaches, and, here, we provide an overview of the methods and findings.

##### Analysis of Macro/Supramolecular Structure

The macro/supramolecular structures can be interrogated through the use of SHG polarization manipulation and analyses. Here, polarization refers to the direction of the electric field vector relative to the direction of the collagen fibers. In one implementation, we obtain the pitch angle of the single α-helical chains, where we define this as the angle of the coil relative to the long-axis collagen molecule. As determined by ultrastructural studies, in normal Col I, this angle is about 47 degrees, and our SHG approach on the tendon (all Col I) is consistent with the result [23].

It had been suggested by immunostaining that the Col III isoform is up-regulated in both HGSOC and benign ovarian tumors. Based on ultrastructural data, and our SHG studies, its corresponding pitch angle is 49 degrees and we investigated if this was borne out in human ovarian tissues [31]. We found that the angles were statistically different between normal, HGSOC and benign tumors, as follows: (i) where the pitch angle in the normal stroma was consistent with Col I; (ii) the angle in benign tumors was consistent with increased Col III; and (iii) HGSOC was different but the angle was lower than normal. This was also borne out throughout our recent mass spectrometry analysis [20]. We further completed a second approach combining SHG with circular dichroism (SHG-CD), which, in an analogy to conventional CD, is sensitive to the chirality of the triple helix [32]. Our results showed that the effective chirality was lower in the HGSOC and benign tumors relative to the normal stroma [31]. Lastly, we performed an analysis of the SHG signal that yielded the net alignment of molecules along the axis of the fibrils. In a normal fibril, the collagen molecules lie essentially on the axis for its length [33]. In contrast, we found that this alignment was highly disrupted in both HGSOC and benign tumors. Collectively, these results indicate that the new desmoplastic collagen is made incorrectly relative to normal Col I.

##### Analysis of Fibril Size and Packing

Unlike fluorescence, which emits isotropically, SHG has a spatial emission pattern that arises from the sub-resolution fibril size and packing. Specifically, the fibril packing results in a distribution of forward (F) and backward (B)-propagating components, which we define as the SHG directionality, F_SHG_/B_SHG_, where larger values arise from greater fibril alignment [34]. We determine this ratio via a combination of measurements and Monte Carlo simulations using optical scattering data [35]. The resulting wavelength dependence of F_SHG_/B_SHG_ can then be used to assess the size and packing. This analysis showed that fibrils in HGSOC were small, more uniformly distributed in diameter and packed more regularly than normal tissues [36]. We obtained similar results by TEM, validating the experimental and theoretical framework. The analogous results on other ovarian tissues (e.g., low-grade and high-risk tissues) are characterized by a higher frequency of large fibrils distributed widely throughout.

### 2.2. Multimodal Imaging Modalities Approaches

Although we have shown in various studies that collagen is significantly altered, both structurally and biochemically, the significance of collagen remodeling in HGSOC progression is still unclear. For example, is it causal or simply characteristic? As cells and many other ECM components (e.g., fibronectin, laminin and Col IV) are transparent to SHG, insight can be gained from multimodal approaches. This will be explored here and is summarized in Table 1.

#### Combined SHG/MPM and MPM/OCT

As SHG does not detect cells, it is important to use other optical modalities that provide cellular context for collagen remodeling. As an example, Sawyer et al. complemented multiphoton fluorescence microscopy (MPM) with wide-field fluorescence imaging (WFI) to acquire high-resolution images of the whole ovary and evaluate the expression of receptors in several genotypes of a mouse ovarian cancer model. Using texture analyses of both the fluorescence and SHG images, they were able to further differentiate between tissues with high statistical significance [37,38]. Similarly, they used optical coherence tomography (OCT) and multiphoton-excited fluorescence to image mouse ovaries in vivo to test the feasibility of using a multimodal approach covering different size scales. They identified and tracked the microscopic changes that occurred early in ovarian cancer development and throughout a mouse’s lifetime, and ultimately determined that this combined approach provided extensive diagnostic, qualitative and quantitative information on the entire mouse ovary [39]. Together, these data show that combining SHG with other imaging techniques significantly improves sensitivity to pathological areas of the ovary.

### 2.3. Optical Scattering and Inverse Spectroscopic Optical Coherence Tomography (ISOCT)

The cellular interactions within the tissue microenvironment during tumor progression are one of the main lines of inquiry in the cancer biology field. However, there still is a need to non-invasively analyze and quantify these interactions within 3D model systems to further delineate physical changes in the TME due to cell–matrix interactions. Optical scattering is an attractive modality for this purpose and has long been used as a tissue characterization tool, where the strength and wavelength dependence is related to the size and distribution of scatterers relative to the wavelength [40,41]. The Backman group has shown how this technique is applicable to objects on the size scale of ~50 nm–1 micron. Moreover, they have used this extensively to characterize colorectal and pancreatic cancers with respect to field carcinogenesis (microarchitectural and microvascular alterations that provide a fertile environment for focal mutations that lead to carcinogenesis) [42,43,44].

We used this theory in conjunction with our implementation of measuring scattering coefficients for the six classes of ovarian tissues probed with texture and polarization analyses and found that the HGSOC tumors were more highly scattering (consistent with desmoplasia) but less organized than normal tissues, with the other tissues (benign, endometrioid, low grade serous) showing intermediate behavior [36].

More recently, Backman showed how this information is encoded in a form of OCT known as inverse scattering OCT (ISOCT), thus providing the collagen/cellular context for the scattering determinations [44]. Spicer et al. examined matrix remodeling in a 3D in vitro model system and showed that cancer cells modify their shape to adjust to a stiffer matrix scaffold due to increased crosslinking of collagen [45]. This further supports the understanding that collagen may enhance the metastatic potential and motility of cells and contribute to poor prognosis and overall survival. The ability to detect and capture early metastases in these systems via ISOCT shows great potential for improving cancer therapeutics and interventions. Lee et al. expanded upon these ideas by utilizing ISOCT and two-photon autofluorescence to quantify nanoscale ultrastructural and metabolic changes, respectively, in ocular surface lesions leading to ocular surface squamous neoplasia (OSSN) [46]. Their results suggested that lesions with increased malignant potential had a higher D (mass density measurement) and a decrease in the optical redox ratio (cancer association via increased cellular proliferation or differentiation), which collectively supported ultrastructural compaction and modifications. Consequently, they were able to differentiate between normal, premalignant and malignant tissue samples more readily [46].

### 2.4. In Vivo Imaging Developments

One of the major challenges of gynecologic cancer research is the lack of imaging modalities capable of reaching and imaging the fallopian tubes and the ovaries, as they are deep within the abdomen and are smaller in size and diameter in comparison to other organs. Currently, microendoscopes capable of meeting this challenge do not exist, where, for example, initial falloposcope designs lacked the ability to fit and traverse the narrow diameter of the FT. They also lack the capability of simultaneously acquiring high-resolution images and collecting fallopian tube epithelial (FTE) cells for future analyses. As a step, Barton and co-workers developed the cell-acquiring fallopian endoscope (CAFE), which met clinical size requirements for eventual in vivo use. This was successfully demonstrated on ex vivo tissues where cells were scraped and then imaged, showing the promise of identification of potentially pathological tissue for subsequent analyses [47]. The Barton lab has further explored various ways to miniaturize imaging systems and components to visualize these changes, and the optimal way to insert the falloposcope into the patient, whether through the uterus and tubal ostium via a standard hysteroscope during surgery or transvaginally [48]. While this work used OCT and widefield fluorescence, integration with SHG is possible with further development. Moreover, they have also pinpointed how much pressure the segments (proximal, middle, distal) of the FT can withstand in order for dilation and allow for endoscopic exploration without bursting or damaging the underlying plicae [49]. While these findings were in porcine FTs, they paved the way for additional endoscopic exploration studies in corresponding human FTs. 

### 2.5. SHG Image-Based Models

**Figure 4:**
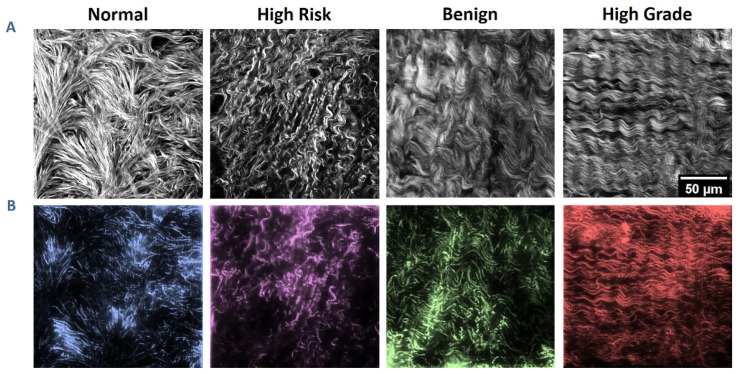
Ovarian stromal images and corresponding fabricated scaffolds. (**A**) SHG optical sections of collagen from the four categories of ovarian tissues. (**B**) Two-photon excited fluorescence images of the resulting respective tissue-engineered scaffolds. Each pattern is 200 × 200 µm in size with 10 µm in height. Scale bar = 50 µm. Reproduced by permission from ref. [51].

As steps to understanding the biological consequences of collagen remodeling, our lab has developed a nano/microfabrication method based on multiphoton-excited polymerization, where we create tissue-engineered scaffolds with designs based on the SHG images. As examples, representative SHG images and the corresponding scaffolds created from a mixture of collagen/gelMA are shown in Figure 4 [50,51]. We have seeded these scaffolds with normal and a series of HGSOC cells and studied aspects of migration, cytoskeletal dynamics and molecular expression of cadherins. This approach allows us to hypothesis test the respective roles of the initial cell phenotype and matrix morphology. In all measurements, we found that the latter dominates the cell response through a contact guidance mechanism. For example, normal cells have greater motility on a cancer matrix than a normal model. This platform will allow detailed biological analysis of cell response as well as an approach to determine treatment efficacy.

## 3. Future Directions/Perspectives

Understanding the changes that occur in the ECM holds the key to developing more efficient and sensitive imaging modalities for early stage detection of ovarian cancer. Our ability to study collagen reorganization and evaluate its role in ovarian cancer progression is a promising piece of a complex puzzle. In this perspective, we have described ways in which imaging collagen and other components of the TME have provided new insights into specific changes in the macro/supramolecular, fibril and fiber architecture in HGSOC. However, we have not yet determined if these changes are predictive or prognostic of HGSOC, as has been shown to be the case in high-grade breast cancer [14]. Ovarian cancers are far less common than those of the breast and we do not yet have the numbers to statistically validate this relationship.

Still, there are paths forward where we can use this type of data to help improve patient health outcomes. For example, a largely unexplored target is correlating collagen alterations with genetic signatures. The prominent genes mutated in HGSOC are BRCA I, BRCA II and Tp53 [52,53,54]. About 5–10% of women have the BRCA I and/or BRCA II mutations [55], while the Tp53 tumor suppressor gene (TSG) is pathogenically mutated in more than 97% of HGSOC diagnoses [56], resulting in a deficient synthesis of the protein and a gain of cancer-promoting features such as increased proliferation, apoptosis evasion, epithelial-to-mesenchymal transition (EMT), and invasion and metastatic behaviors. However, there is still much uncertainty about how aberrant p53 is involved in facilitating cellular interactions with remodeled collagen. In order to understand this, we must consider both the indirect and direct roles wildtype p53 has on ECM and ECM-related gene expression patterns, specifically on collagen synthesis, maturation, and activation. As a first step, we showed that the distribution of the SHG emission pattern was more heterogeneous for low-grade disease than HGSOC, and postulated that this was due to the broader range of mutations in the former [57].

We also foresee a couple of possibilities related to determining therapeutic efficacy based on the SHG data. The standard of care is to remove the ovary either before or after chemotherapy. That tissue could be imaged and then its structure correlated with survival following chemotherapy. The primary metastatic site is the omentum [58,59,60], and most deaths occur from this metastasis and other sites within the peritoneal cavity, so this is still a viable strategy even with the ovary being removed. Analogously, we could image removed tissues and create imaged-based models as described in Section 2.5, and seed with these with a patient’s cells collected from ascites and then treat with combinations of chemotherapies to predict the best course of treatment.

**Figure 5:**
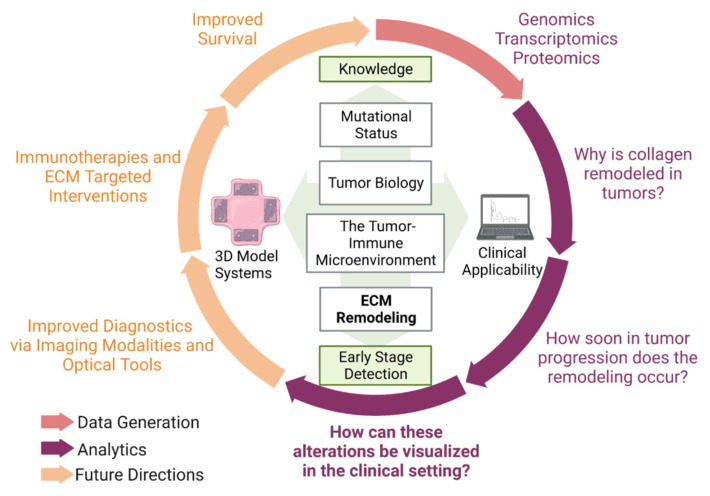
Summary of research perspective. This perspective expanded upon the significance of ECM modifications in ovarian cancer and the optical tools that have been utilized to visualize these alterations. However, there are other considerations to improve diagnostics and survival statistics. Figure created with biorender.com.

While in vitro experiments have shown that increased collagen density affected several signaling pathways, these measurements were not performed in tissues with known mutations [61,62] (and references therein) and were not imaged by SHG. Burdette and co-workers have developed several FT lines with known mutations that form tumors in vivo, and, moreover, the migration/invasion in vitro was sensitive to the collagen microenvironment [63,64,65]. We suggest that imaging these and related tissues by SHG and other high-resolution optical methods described in this perspective will provide new insight into the etiology and progression of HGSOC. These concepts are depicted in Figure 5. We propose that in vivo and in vitro experiments with HGSOC tumors whose mutations are defined using genomics, transcriptomics and proteomic approaches can be used to study the molecular and cellular parameters that initiate collagen remodeling as determined by SHG imaging. This information will be critical to defining the earliest time interval when collagen remodeling occurs in the progression of HGSOC. Imaging approaches can be developed and employed to specifically identify these early changes in collagen with the goal of developing novel diagnostic tools for early detection of HGSOC. In addition to diagnosis, the understanding of the structural modifications in collagen in early and advanced HGSOC can be used to develop specific targeted therapies (for example, antibodies or engineered T cells that recognize disordered collagen) as means for treatment of the cancer leading to increased survival of women with HGSOC. We postulate that successful diagnosis/treatment of HGSOC requires an integrated approach of ex vivo microscopic and molecular analyses and the use of in vitro models to establish the foundation for in vivo imaging.

A second area with significant potential is the development of high-resolution optical tools that can be used in vivo. While their development is still somewhat nascent, this goal has great merit, as we have shown optical changes in both the ovary and FT that coincide with pathology. Notably, cellular changes in precursors are subtle and difficult to identify, even by a trained pathologist. In contrast, collagen changes in p53 precursors and STICs were readily revealed by SHG and accurately classified by image analysis. Given that it is likely these precursors require several years to develop into high-grade disease, an SHG-based microendoscope could be a viable approach for screening patients at high risk of developing HGSOC. Thus, the development of an imaging tool that is compact, flexible, sensitive, steerable, non-ionizing and minimally invasive would significantly improve our ability to detect these lesions and diagnose ovarian cancer in the early stages. Many of these tools described in this perspective could serve as the foundation for the development of this endoscopy technology.

## 4. Conclusions

In this perspective, we described the limitations of existing imaging and screening techniques for the detection of HGSOC. To address this problem, we described the use of high-resolution SHG imaging of collagen alterations in the ECM as a means to identify early changes in this disease, where we can delineate between precursors in the fallopian tubes and frank tumors therein, as well as between HGSOC, other ovarian tumors and normal tissues. For a more complete characterization of the ECM changes, we also described how SHG can be combined with other modalities including fluorescence and optical coherence tomography (OCT). For eventual direct translation to clinical imaging, SHG will need to be implemented via a microendoscope and we describe the state of the art in these developments. We postulate that successful diagnosis/treatment of HGSOC requires an integrated approach of ex vivo microscopic and molecular analyses to establish the foundation for in vivo imaging.

## Figures and Tables

**Table 1:** Overview of imaging modalities. This table summarizes the typical imaging modalities used in the basic and clinical research settings. While each individual modality has its advantages and disadvantages, there is still useful information provided, especially when combined with other imaging tools.

Imaging Technique	How It Works	Measurements	Advantages	Disadvantages	Setting
Second Harmonic Generations (SHG) Microscopy	Nonlinear coherent up-conversion of two lower-energy photons into one higher energy photon	Collagen fibril/fiber organization in tissues	-No exogenous dyes needed -Detects inherent fluorescence of collagen molecule -High-resolution images of collagen fibers	-Transparent to cells -Specific to collagen I, cannot detect other collagen types or other ECM proteins -Signal dependent on collagen density	Mouse, pre-clinical
Multiphoton Fluorescence Microscopy (MPM)	Laser scanning + long wavelength excitation	Biological processes in living cells and tissues	-Low negative impact on cell/tissue viability -Provides 3-dimensional view -Near-infrared excitation allows for deep penetration into biological specimen	-Limited sensitivity, excitation occurs only at focal point of microscope -Must use fluorophores to tag molecule(s) of interest	Mouse, pre-clinical
Optical Coherence Tomography (OCT)	Coherent backscattered light from bulk tissue sample	Cross-sectional tissue morphology	-Provides overview of tissue architecture	-1–10 µm microscopic resolution -Cannot detect nanoscale structures (i.e., microvasculature alterations such as collagen alterations)	Mouse, pre-clinical, clinical (eye)
Inverse Spectroscopic Optical Coherence Tomography (ISOCT)	Tissue modeled as a medium with a continuously varying refractive index (RI)	Collagen, cellular content, biological media	-Sensitive to tissue ultrastructure, can provide quantitative information -detection range 30–450 nm -Can also be used to evaluate blood vessels	-Based solely on scattering properties of samples -The inverse of OCT measurements	Mouse, pre-clinical
